# Malnutrition Is Highly Prevalent in Patients With Chronic Pancreatitis and Characterized by Loss of Skeletal Muscle Mass but Absence of Impaired Physical Function

**DOI:** 10.3389/fnut.2022.889489

**Published:** 2022-06-01

**Authors:** Mats L. Wiese, Simone Gärtner, Nele von Essen, Julia Doller, Fabian Frost, Quang Trung Tran, Frank Ulrich Weiss, Fatuma Meyer, Luzia Valentini, Leif-A. Garbe, Cornelia C. Metges, Karen Bannert, Lea Franziska Sautter, Luise Ehlers, Robert Jaster, Georg Lamprecht, Antje Steveling, Markus M. Lerch, Ali A. Aghdassi

**Affiliations:** ^1^Department of Medicine A, University Medicine Greifswald, Greifswald, Germany; ^2^Department of Internal Medicine, Hue University of Medicine and Pharmacy, Hue University, Hue, Vietnam; ^3^Institute of Evidence-Based Dietetics (NIED), University of Applied Sciences Neubrandenburg, Neubrandenburg, Germany; ^4^Department of Agriculture and Food Sciences, University of Applied Sciences Neubrandenburg, Neubrandenburg, Germany; ^5^Institute of Nutritional Physiology “Oskar Kellner”, Research Institute for Farm Animal Biology (FBN), Dummerstorf, Germany; ^6^Department of Medicine II, Division of Gastroenterology, Rostock University Medical Center, Rostock, Germany; ^7^Ludwig Maximilian University Hospital, Ludwig Maximilian University of Munich, Munich, Germany

**Keywords:** malnutrition, chronic pancreatitis, sarcopenia, GLIM, handgrip strength

## Abstract

**Background/Aims:**

Patients with chronic pancreatitis (CP) have an increased risk of malnutrition, a condition linked to reduced muscle mass and physical performance. We have investigated the risk factors, phenotypic presentation, and health implications associated with malnutrition in CP.

**Materials and Methods:**

In a multicenter cross-sectional study we recruited patients with confirmed CP and healthy volunteers as a control group. Malnutrition was diagnosed according to the criteria proposed by the Global Leadership Initiative on Malnutrition. We performed detailed examinations of body composition and physical function as well as testing of routine blood parameters and markers of inflammation.

**Results:**

We included 66 patients [mean (±SD) age: 56.0 (±14.5) years; 51 males] and an equal number of age- and sex-matched controls. Moderate malnutrition was diagnosed in 21% (*n* = 14) and severe malnutrition in 42% (*n* = 28) of patients. Besides weight loss malnourished patients showed lower fat and skeletal muscle mass compared to both non-malnourished subjects and healthy controls. Only in severe malnutrition, blood parameters reflected elevated inflammation and reduced muscle reserves. Handgrip strength in patients did not differ by nutritional status but there was a significant correlation (rho = 0.705, *p* < 0.001) with skeletal muscle mass. Although 20 patients (30%) had pathologically reduced skeletal muscle mass, only two individuals (3%) had sarcopenia with concomitantly reduced handgrip strength.

**Conclusion:**

Malnutrition is a frequent complication of CP characterized by loss of skeletal muscle mass. As this condition becomes evident only at an advanced stage, regular testing for altered body composition is recommended. Suitable biomarkers and the link between loss of muscle mass and physical function require further investigation.

**Clinical Trial Registration:**

[https://clinicaltrials.gov/ct2/show/NCT04474743], identifier [NCT04474743].

## Introduction

Chronic pancreatitis (CP) is a fibro-inflammatory disease of the pancreas characterized by a gradual decline of pancreatic exocrine and endocrine function secondary to progressive fibrosis (1, 2). This loss of pancreatic function imposes a significant risk to the patient’s nutritional status as nutrient digestion and uptake will eventually become compromised. As a consequence, malnutrition is a common complication of CP which is associated with increased morbidity and mortality (3, 4).

In the past, different parameters have been applied to define a state of malnutrition in CP, not always mutually interchangeable because they refer to different nutrition concepts. For instance, when diagnosed by body mass index (BMI), reported prevalence ranges from 8 to 39% (5, 6). When considering fat-soluble vitamin deficiency numbers vary even more, from 1 to 35% and from 33 to 87% for vitamin A (4, 7–9) and vitamin D (4, 8, 9), respectively. Use of these inconsistent definitions hampers both an accurate estimation of the prevalence and the identification of risk factors associated with malnutrition in CP. Moreover, the health consequences of malnutrition, for instance its relation with sarcopenia and disease prognosis, are not well studied (10, 11).

Assessment of nutritional status based on BMI alone has limitations and is nowadays considered inadequate to assess nutritional status in patients with CP (2, 12). Low muscle mass, which has been linked to adverse health outcome in various disease states, is now commonly included in diagnostic criteria for malnutrition (13). However, clear recommendations on suitable methods and parameters to define this condition in patients with CP are still missing. In consequence, diagnosis of malnutrition, especially at an early stage, remains challenging for the clinician and initiation of nutrition therapy may be delayed.

In 2018, the Global Leadership Initiative on Malnutrition (GLIM) for the first time published global consensus criteria for the diagnosis of malnutrition in clinical settings (14). Application of these criteria could harmonize research on malnutrition in CP and thereby add to filling the above-mentioned research gaps. To the best of our knowledge, this is the first study to prospectively apply the GLIM criteria in a cohort of patients with CP. It comprises a detailed characterization of nutritional status in patients with CP. By additionally examining the risk factors, phenotypic presentation, and health implications associated with malnutrition, we aim to provide guidance for daily clinical practice.

## Materials and Methods

### Study Design and Population

In this multicentric cross-sectional prospective study, patients with chronic pancreatitis were enrolled at University Medicine Greifswald and Rostock University Medical Center, two tertiary referral centers in Northeast Germany. As a control group, healthy volunteers were recruited from the general public in the city of Neubrandenburg, which is located in the same region. Recruitment took place between October 2018 and September 2021. The study was approved by the respective Local Institutional Review Boards (internal registration numbers: A 2018-0129, BB 155/18, HSNB/AL/143/18) and registered at clinicaltrials.gov (NCT04474743). All methods were carried out in accordance with the relevant guidelines and regulations (Declaration of Helsinki).

All patients 18 years or older with chronic pancreatitis confirmed by imaging modality, based on either endoscopic ultrasound, computed tomography, or magnetic resonance imaging with magnetic resonance cholangiopancreatography and/or histology were screened for study participation. Patients were considered eligible if none of the following exclusion criteria was met: (1) diagnosis of a malignant disease within the last 3 years, (2) pregnancy or lactation, (3) concomitant other severe chronic gastrointestinal disease, including liver cirrhosis, or (4) relevant cognitive and/or physical restraints. Healthy volunteers by definition had to be free of acute or chronic disease. In addition, weight stability within the last 6 months, a BMI between 18.5 and 34.9 kg/m^2^ and a good state of health and performance, equivalent to an Eastern Cooperative Oncology Group Status of 0, were mandatory in this group.

### Data Collection

#### Clinical and Patient Data

Personal and disease-related data were obtained by standardized interview or extracted from the patient files. Disease severity was graded using Chronic Pancreatitis Prognosis Score (COPPS), a validated scoring system to predict short-term prognosis in patients with CP (15). For assessment of exocrine pancreatic function, we measured levels of fecal elastase-1 in all patients. The established cut-off of 200 μg/g was set to define exocrine insufficiency, with values below 100 μg/g indicating severe impairment. Pancreatogenic diabetes was diagnosed based on the presence of major and minor criteria as suggested by Ewald and Bretzel (16). Dietary intake was assessed by a validated semi-quantitative food frequency questionnaire (17) inquiring patients’ food consumption of the 4 weeks prior to hospital attendance. The short form of the International Physical Activity Questionnaire (18) was used to determine physical activity during the last 7 days preceding attendance.

#### Physical Examination and Blood Testing

Anthropometric measurements were performed in patients and controls following a standardized protocol with quality assurance across centers and included the following parameters: body weight and height, waist, hip and mid-upper arm circumference as well as triceps skinfold thickness. At all study centers, body composition was analyzed with the seca mBCA 515 (seca, Hamburg, Germany), an eight-electrode, phase-sensitive, segmental bioelectrical impedance analysis (BIA) device. Handgrip strength was tested employing the Jamar Plus+ Digital Hand Dynamometer (Patterson Medical, Warrenville, IL, United States). Three measurements were taken with the patients seated, the elbow in 90° flexion, and the wrist in a neutral position using their dominant hand. The maximum value of the three attempts was considered for analysis. For additional assessment of physical performance, patients and controls completed 4-m gait speed test. Blood samples were drawn from all subjects. Besides routine blood parameters, we determined markers associated with inflammation or nutritional status.

#### Diagnosis of Malnutrition and Sarcopenia

Malnutrition was diagnosed according to the GLIM criteria. A detailed report of the criteria is provided elsewhere (14). Briefly, the diagnosis of malnutrition according to GLIM requires the combination of at least one phenotypic and one etiologic criterion. Because CP is characterized by recurrent inflammatory episodes, the etiologic criterion of inflammation was considered present in all patients. Phenotypic criteria were used for severity grading into moderate and severe malnutrition. For BMI and weight loss, the thresholds as suggested by GLIM were applied. Because of absence of established cut-offs for total skeletal muscle mass (SMM) for delineation of malnutrition assessed by BIA, we referred to device-specific reference values obtained from a sample of healthy blood donors aged 18–65 years and representative for the German population (19). Sex-specific thresholds for skeletal muscle mass index (SMMI), i.e., SMM divided by height^2^, were defined as mean minus one standard deviation (♀: 6.68 kg/m^2^, ♂: 8.97 kg/m^2^) or mean minus two standard deviations (♀: 5.86 kg/m^2^, ♂: 8.14 kg/m^2^) for moderate and severe malnutrition, respectively.

Sarcopenia was diagnosed according to the European Working Group on Sarcopenia in Older People 2 (EWGSOP2) criteria (20). For confirmation of sarcopenia in patients with reduced handgrip strength (♀: <16 kg, ♂: <27 kg) the same cut-offs for SMM as for diagnosis of severe malnutrition were applied. Sarcopenia was defined severe in patients slower than 0.8 m/s in the 4-m gait speed test.

### Statistical Analysis

Descriptive data is presented as mean (±SD) or median (IQR) for normally and non-normally distributed continuous variables, respectively; categorical data is presented as n (%). To compare continuous variables between groups, two-tailed *t*-test or Mann–Whitney-*U* test were employed as indicated by the distribution of the data tested. Chi-squared test or Fisher’s exact test were used to examine differences in categorical variables. Spearman’s correlation coefficient was calculated to measure the strength and direction of association between SMMI and handgrip strength. A *p*-value of less than 0.05 was defined as statistically significant. For comparison between patients and healthy controls, subjects were matched by age and sex in a 1:1 ratio for each nutritional status subgroup. All statistical analyses were performed using IBM SPSS Statistics for Windows, version 27 (IBM Corp., Armonk, NY, United States). For matching and graphical visualization of results we employed R software (R Core Team, Vienna, Austria) for statistical computing (version 4.1.0) (21).

## Results

### Patient Selection and Characteristics

A total of 80 patients with definite CP and 94 healthy controls were prospectively recruited for this study. Subjects with incomplete data or detection of an *a priori* exclusion criterion after study enrollment were excluded from the analyses. Among the remaining 66 patients, 42 (64%) were malnourished, with 28 (42%) having severe and 14 (21%) moderate malnutrition. Table 1 summarizes key patient characteristics stratified by nutritional status.

**TABLE 1:** Demographic and clinical characteristics of patients with chronic pancreatitis stratified by nutritional status.

	No malnutrition (*n* = 24)	Moderate malnutrition (*n* = 14)	Severe malnutrition (*n* = 28)	
Age, years^a^	53.5 (±15.9)	56.4 (±13.8)	58.1 (±13.8)	
Male, n (%)	16 (67)	12 (86)	23 (82)	
Outcare treatment, n (%)	16 (67)	8 (57)	14 (50)	
**Etiological risk factors, n (%)**				
Alcohol	8 (33)	5 (36)	14 (50)	
Nicotine	13 (54)	9 (64)	20 (71)	
Autoimmune	–	1 (7)	2 (7)	
Idiopathic	16 (67)	8 (57)	12 (43)	
Genetic risk factors	6 (25)	5 (36)	6 (21)	
**Continued substance abuse, n (%)**				
Alcohol	7 (29)	5 (36)	10 (36)	
Nicotine	9 (38)	5 (36)	18 (64)	
Exocrine pancreatic insufficiency, n (%)	9 (38)	7 (50)	15 (54)	
Fecal elastase <100 μg/g	6 (25)	3 (21)	13 (46)	
Untreated	3 (33)	3 (43)	5 (33)	
Pancreatic enzyme replacement therapy, n (%)	9 (38)	8 (57)	16 (57)	
Endocrine pancreatic insufficiency, n (%)	4 (17)	–	5 (18)	
Pancreatic surgery, n (%)	3 (13)	2 (14)	5 (18)	
Endoscopic intervention, n (%)	5 (21)	6 (43)	10 (36)	
Opioid treatment, n (%)	3 (13)	–	6 (21)	
NRS Pain (0–10)^b^	3 (5)	0 (3)	2.5 (6)	†
Diagnosis within last 12 months, n (%)	9 (38)	3 (21)	18 (64)	#
**COPPS, n (%)**				* ‡
A	12 (50)	4 (29)	4 (14)	
B	11 (46)	8 (57)	16 (57)	
C	1 (4)	2 (14)	8 (29)	
NRS-2002 ≥3, n (%)	1 (4)	3 (21)	15 (54)	* ‡ #

*COPPS, Chronic Pancreatitis Prognosis Score; NRS-2002, Nutritional Risk Screening 2002; NRS Pain, Numeric rating scale for pain: 0 = no pain, 10 = worst imaginable pain (within past 7 days).*

*^a^Data is presented as mean (±SD);*

*^b^Data is presented as median (IQR).*

*Differences between groups were tested using Chi-squared test or Fisher’s exact test for categorical variables and two-tailed t-test for or Mann-Whitney-U test for continuous variables.*

*Indicates significant difference between patients with and without malnutrition, p < 0.05.

*^†^Indicates significant difference between patients without malnutrition and with moderate malnutrition, p < 0.05.*

*^‡^Indicates significant difference between patients without malnutrition and with severe malnutrition, p < 0.05.*

*^#^Indicates significant difference between patients with moderate and with severe malnutrition, p < 0.05.*

Nutritional status was independent of age, sex, or any etiologic risk factor. Moreover, the percentage of outpatients was similar among the three groups. While there was also no relation to continued alcohol consumption, ongoing nicotine abuse, although statistically non-significant, tended to be more common in malnourished patients, especially in those with a severe grade (*p* = 0.054). Malnutrition was neither associated with pancreatic exocrine nor endocrine function (*p* = 0.525 and *p* = 0.713, respectively). We saw a similar prevalence of exocrine pancreatic insufficiency (EPI), between malnourished and non-malnourished patients and the same applied to severe exocrine impairment. In addition, there were no significant differences regarding the percentage of patients on pancreatic enzyme replacement therapy. Approximately, one in three patients had not received pancreatic enzyme replacement therapy before diagnosis of EPI but numbers were comparable between malnourished and non-malnourished subjects. Having had pancreatic surgery and treatment with opioids were not linked with nutritional status either.

Significant associations with nutritional status were seen with disease severity and duration. While 50% of non-malnourished patients had COPPS A, the majority of moderately and severely malnourished patients (71 and 86%, respectively) were classified as COPPS B or C (*p* = 0.015). Severe malnutrition was more common in patients with disease duration of 12 months or less. Among severely malnourished subjects, 64% had received diagnosis of CP within the last year as compared to 38% (*p* = 0.054) and 21% (*p* = 0.009) in non- and moderately malnourished subjects, respectively.

Finally, malnutrition diagnosis was significantly associated with a positive screening result on the NRS-2002 (*p* = 0.001). However, there was no difference between moderately and non-malnourished patients (*p* = 0.132). Overall, the NRS-2002 showed high specificity (96%) but low sensitivity (43%) for detection of malnutrition.

### Prevalence of Etiologic and Phenotypic Criteria

The prevalence of etiologic and phenotypic criteria for malnutrition according to GLIM differed between groups with no, moderate, or severe malnutrition (Table 2). Malnourished patients reported reduced food intake more often than non-malnourished subjects. Frequencies of food intake being 50% or less of energy requirements as well as any reduced intake for longer than 2 weeks varied by nutritional status (*p* = 0.002 and *p* = 0.012, respectively). By contrast, neither the presence of chronic gastrointestinal conditions adversely impacting food assimilation or absorption, nor the state of acute disease or injury differed significantly between these three groups (*p* = 0.727 and *p* = 0.116, respectively).

**TABLE 2:** Prevalence of etiologic and phenotypic GLIM criteria among patients with chronic pancreatitis.

	No malnutrition (*n* = 24)	Moderate malnutrition (*n* = 14)	Severe malnutrition (*n* = 28)	
**Etiologic criteria**				***p*-value^$^**
*Reduced food intake or assimilation*				
≤50% of energy requirements for longer than 1 week	3 (13)	3 (21)	16 (57)	0.002
Any reduction for longer than 2 weeks	3 (13)	2 (14)	13 (46)	0.012
Chronic gastrointestinal condition adversely impacting food assimilation or absorption	9 (38)	7 (50)	12 (43)	0.727
*Disease burden/inflammation*				
Acute disease/injury	2 (8)	3 (21)	9 (32)	0.116
Chronic disease-related	24 (100)	14 (100)	28 (100)	–
**Phenotypic criteria**				***p*-value^‡^**
*Low body mass index, n (%)^1^*				0.085
N/A	24 (100)	14 (100)	19 (68)	
Moderate	–	–	5 (18)	
Severe	–	–	4 (14)	
*Weight loss, n (%)^2^*				<0.001
N/A	24 (100)	10 (71)	6 (21)	
Moderate	–	4 (29)	2 (7)	
Severe	–	–	20 (71)	
*Reduced muscle mass, n (%)^3^*				<0.001
N/A	24 (100)	2 (14)	5 (18)	
Moderate	–	12 (86)	3 (11)	
Severe	–	–	20 (71)	

*^$^Differences between groups of nutritional status were tested using Chi-squared test or Fisher’s exact test, respectively.*

*^‡^Differences between patients with moderate and severe malnutrition were tested using Fisher’s exact test.*

*^1^Moderate: <20 kg/m^2^ if age >70 years or 22 kg/m^2^ if age ≥70 years; severe: <18.5 kg/m^2^ if age >70 years or 20 kg/m^2^ if age ≥70 years.*

*^2^Moderate: 5–10% within the past 6 months, or 10–20% beyond 6 months; severe: >10% within the past 6 months, or >20% beyond 6 months.*

*^3^Moderate: Skeletal muscle mass index <8.97 kg/m^2^ if male or, <6.68 kg/m^2^ if female; severe: Skeletal muscle mass index <8.14 kg/m^2^ if male, or <5.86 kg/m^2^.*

In addition, the patterns of phenotypic GLIM criteria were slightly different between grades of malnutrition. Moderate malnutrition was primarily characterized by reduced muscle mass and – to a lesser extent – by weight loss (86 and 29% of patients, respectively) while low body mass index was not found in any patient. Contrary to this, reduced muscle mass and weight loss were similarly distributed in severe malnutrition (71% of patients). Additionally, 18 and 14% of severely malnourished patients presented with moderately or severely lowered BMI, respectively.

### Anthropometric Parameters and Body Composition

**FIGURE 1:**
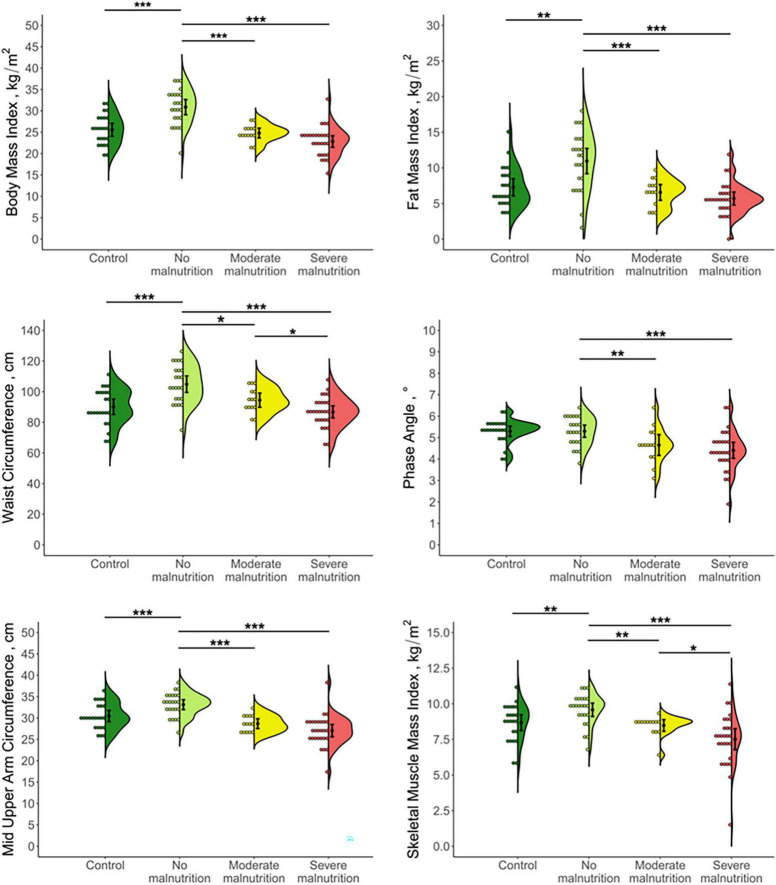
Jellyfish plots of anthropometric and body composition parameters in patients with chronic pancreatitis stratified by nutritional status and healthy controls. Distribution of data is presented as a combination of histogram (left), mean ± SD (center), and kernel density estimated probability density function (right). Differences between groups were tested by two-tailed *t*-test. *p < 0.05, **p < 0.01, ***p < 0.001.

There were distinct differences in both anthropometric and body composition parameters between malnourished and non-malnourished patients (Figure 1). Non-malnourished patients presented an incipient obesity phenotype characterized by a higher BMI, fat mass index, and waist circumference than age- and sex-matched controls. On the other hand, malnourished patients were characterized by lower fat reserves and reduced muscle mass compared to non-malnourished subjects. In severely malnourished patients, almost all anthropometric and body composition parameters differed from healthy controls. By contrast, in moderately malnourished patients only hip and mid upper arm circumference as well as phase angle were diminished (Supplementary Table 1). Although almost all anthropometric and body composition parameters could distinguish between malnourished and non-malnourished patients, only waist and hip circumference as well as SMMI significantly differed in moderate and severe malnutrition (Table 3).

**TABLE 3:** Comparison of anthropometric parameters and body composition in patients with chronic pancreatitis stratified by nutritional status.

	No malnutrition (*n* = 24)	Moderate malnutrition (*n* = 14)	Severe malnutrition (*n* = 28)	
Body weight, kg	92.3 (±11.5)	77.3 (±9.3)	67.8 (±13.7)	* ^†^ ^‡ #^
Waist circumference, cm	104.9 (±12.7)	94.5 (±8.0)	86.8 (±10.1)	* ^†^ ^‡ #^
Hip circumference, cm	107.8 (±10.8)	97.6 (±4.1)	92.2 (±6.5)	* ^†^ ^‡ #^
Waist-to-Hip ratio	0.98 (±0.11)	0.97 (±0.06)	0.94 (±0.08)	
Mid upper arm circumference, cm	33.1 (±2.7)	28.7 (±2.0)	27.0 (±3.7)	* ^†^ ^‡^
Triceps skinfold thickness, mm	25.2 (±8.4)	14.5 (±3.5)	13.8 (±6.0)	* ^†^ ^‡^
Body mass index, kg/m^2^	30.9 (±4.2)	24.8 (±2.0)	22.8 (±3.5)	* ^†^ ^‡^
Fat mass index, kg/m^2^	11.0 (±4.2)	6.6 (±1.9)	5.7 (±2.3)	* ^†^ ^‡^
Fat free mass index, kg/m^2^	19.9 (±2.0)	18.3 (±1.8)	17.1 (±2.8)	* ^†^ ^‡^
Skeletal muscle mass index, kg/m^2^	9.6 (±1.1)	8.5 (0.7)	7.5 (±1.9)	* ^†^ ^‡ #^
Phase angle,°	5.3 (±0.7)	4.7 (±0.8)	4.4 (±1.0)	* ^†^ ^‡^
Total body water, l	44.4 (±6.3)	41.9 (±5.0)	37.5 (±8.7)	* ^‡^
Extracellular body water, l	19.5 (±2.6)	18.6 (±2.8)	17.2 (±3.3)	* ^‡^
Extracellular to total body water ratio*^a^*	0.43 (0.04)	0.45 (0.04)	0.45 (0.05)	

*All data is presented as mean (±SD) unless indicated otherwise.*

*^a^Data is presented as median (IQR).*

*Differences between groups were tested using two-tailed t-test for or Mann–Whitney-U test for normally and non-normally distributed data, respectively.*

*Indicates significant difference between patients with and without malnutrition, p < 0.05.

*^†^Indicates significant difference between patients without malnutrition and with moderate malnutrition, p < 0.05.*

*^‡^Indicates significant difference between patients without malnutrition and with severe malnutrition, p < 0.05.*

*^#^Indicates significant difference between patients with moderate and with severe malnutrition, p < 0.05.*

### Muscle Strength and Sarcopenia

**FIGURE 2:**
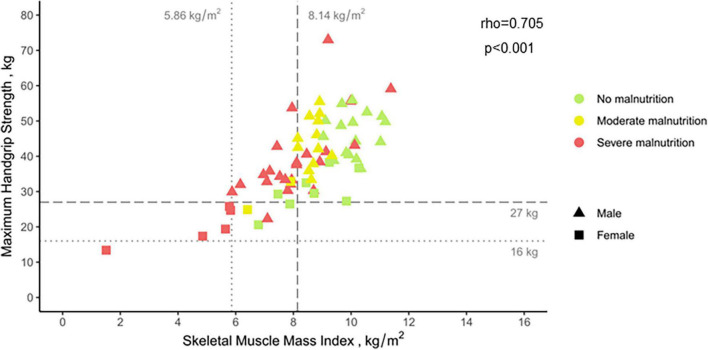
Correlation between skeletal muscle mass index and maximum handgrip strength in patients with chronic pancreatitis. Data is presented stratified by nutritional status and sex. Vertical and horizontal lines indicate sex-specific thresholds of sarcopenia for skeletal muscle mass and handgrip strength, respectively. Spearman’s correlation coefficient was calculated to measure the strength and direction of association between the two variables.

No differences in handgrip strength were found between the different groups of our study cohort. Only in malnourished patients, handgrip strength was reduced in comparison to healthy controls [mean (±SD): 38.0 (±11.9) kg vs. 44.8 (±13.1) kg, *p* = 0.014]. While muscle strength of moderately malnourished patients was similar to controls [mean (±SD): 42.1 (±8.6) kg vs. 44.6 (±13.1) kg, *p* = 0.556], a significant reduction was seen for severely malnourished subjects [mean (±SD): 35.9 (±12.9) kg vs. 44.9 (±14.0) kg, *p* = 0.016]. There was a significant correlation (rho = 0.705, *p* < 0.001) between SMMI and handgrip strength in patients with CP. Only the latter inversely correlated with age (*r* = −0.37, *p* = 0.006). Although 20 subjects (30%) had SMM below the cut-off for sarcopenia, only two patients (3%) showed concomitantly reduced handgrip strength and were thus diagnosed with sarcopenia (Figure 2). In both subjects, sarcopenia was not graded as severe indicated by normal gait speed (>0.8 m/s).

### Blood Parameters

When we compared findings of routine blood parameters in CP patients with different stages of malnutrition including C-reactive protein (CRP), interleukins 6 and 1β as well as tumor necrosis factor α (Table 4), we found that only two routine parameters significantly differed between malnourished and non-malnourished patients: While mean corpuscular volume was higher in malnourished patients, levels of cholinesterase were reduced (*p* = 0.026 and *p* < 0.001, respectively). These differences essentially resulted from deviations between severely and non-malnourished subjects. However, there was a trend toward lower levels of cholinesterase in moderately malnourished patients (*p* = 0.088). In the subgroups of severely and non-malnourished individuals, further differences were observed for several other blood markers. While levels of creatinine, blood urea nitrogen and uric acid were reduced, we found elevated mean corpuscular volume in patients with severe malnutrition. Furthermore, lower concentrations of mean corpuscular hemoglobin and uric acid distinguished severe from moderate grade malnutrition. Regarding serum markers for inflammation, we only detected a significant elevation of CRP in patients with severe malnutrition. However, this marker was not useful for identification of individuals with only moderate malnutrition when compared to CP patients without nutritional deficiency. All other inflammatory markers we investigated were not associated with any stage of malnutrition in CP.

**TABLE 4:** Comparison of blood parameters in patients with chronic pancreatitis stratified by nutritional status.

	Missing data, n (%)	No malnutrition (*n* = 24)	Moderate malnutrition (*n* = 14)	Severe malnutrition (*n* = 28)	
**Complete blood Count**					
Hemoglobin, mmol/L	–	8.5 (±1.4)	8.4 (±1.0)	8.1 (±1.2)	
Hematocrit, L/L	–	0.406 (±0.059)	0.403 (±0.042)	0.396 (±0.054)	
Mean corpuscular volume, fL	–	87.0 (±6.3)	89.0 (±5.0)	90.6 (±5.3)	* ^‡^
Mean corpuscular hematocrit, fmol	–	1.81 (±0.15)	1.86 (±0.11)	1.85 (±0.12)	
Mean corpuscular hemoglobin concentration, mmol/L	–	20.8 (±0.9)	20.9 (±0.5)	20.4 (±0.6)	**^#^**
White blood cell count, 10^9^/L^a^	–	7.4 (2.1)	7.0 (1.9)	8.4 (4.4)	
Platelet count, 10^9^/L^a^	–	237 (62)	204 (83)	237 (129)	
**Blood chemistry**					
Creatinine, μmol/L^a^	–	75 (28)	82 (26)	63 (28)	**^‡^**
Cholinesterase, kU/L	–	13.1 (±2.1)	11.4 (±3.2)	10.1 (±3.2)	* ^‡^
Total bilirubin, μmol/L^a^	–	7.9 (3.3)	7.5 (4.2)	7.7 (5.6)	
Blood urea nitrogen, mmol/L^a^	1 (2)	5.7 (2.3)	4.7 (3.5)	3.9 (2.2)	**‡**
Uric acid, μmol/L^a^	–	322 (107)	318 (119)	275 (131)	**‡ ^#^**
Albumin, g/L^a^	–	38 (6)	39 (6)	37 (11)	
Prealbumin, g/L	1 (2)	0.261 (±0.070)	0.263 (±0.069)	0.228 (±0.086)	
C-reactive protein, mg/L^a^	–	3.1 (4.7)	3.1 (1.7)	5.4 (27.5)	**‡ ^#^**
Interleukin 6, pg/ml^a^	–	2.3 (4.7)	2.6 (3.1)	2.0 (6.8)	
Interleukin 1 beta, pg/ml^a^	4 (6)	4.9 (3.0)	2.0 (3.0)	5.0 (3.3)	
Tumor necrosis factor alpha, pg/ml^a^	3 (4)	5.4 (4.1)	5.9 (2.0)	8.1 (4.7)	

*All data is presented as mean (±SD) unless indicated otherwise.*

*^a^Non-normally distributed data is presented as median (IQR).*

*Differences between groups were tested using two-tailed t-test for or Mann–Whitney-U test for normally and non-normally distributed data, respectively.*

*Indicates significant difference between patients with and without malnutrition, p < 0.05.

*^‡^Indicates significant difference between patients without malnutrition and with severe malnutrition, p < 0.05.*

*^#^Indicates significant difference between patients with moderate and with severe malnutrition, p < 0.05.*

#### Dietary Intake and Physical Activity

In patients with CP, we found significant differences in intake of energy and macronutrients depending on their nutritional status (Table 5). Malnourished patients reported higher intakes of energy and carbohydrates while intakes of protein, dietary fiber, fat, or alcohol did not differ between groups. Subgroup analysis showed that these differences existed only between severely malnourished and non-malnourished patients. Except for alcohol consumption, there were no differences between patients and their age- and sex-matched controls. Irrespective of nutritional status, patients consumed significantly less alcohol than respective controls (Supplementary Table 2).

**TABLE 5:** Comparison of energy and macronutrient intake in patients with chronic pancreatitis stratified by nutritional status.

	No malnutrition (*n* = 23)^a^	Moderate malnutrition (*n* = 14)	Severe malnutrition (*n* = 28)	
Energy, kcal/d	1576 (1421)	2024 (1082)	2171 (1002)	* ^‡^
Protein, g/d	59 (43)	83 (36)	78 (34)	
Carbohydrates, g/d	181 (136)	208 (143)	253 (100)	* ^‡^
Dietary fiber, g/d	21 (18)	20 (14)	23 (13)	
Fat, g/d	55 (61)	88 (45)	78 (50)	
Alcohol, g/d	0 (4)	1 (6)	0 (2)	

*All data is presented as median (IQR).*

*^a^One patient did not complete the food frequency questionnaire and was excluded from analysis.*

*Differences between groups were tested using Mann–Whitney-U test.*

*Indicates significant difference between patients with and without malnutrition, p < 0.05.

*^‡^Indicates significant difference between patients without malnutrition and with severe malnutrition, p < 0.05.*

In terms of physical activity, groups of nutritional status showed a different distribution among activity categories (Table 6). In general, there was a trend toward a higher activity in malnourished patients compared to non-malnourished patients (*p* = 0.057). Moderately malnourished patients reported the highest physical activity, which was significantly elevated compared to subjects without malnutrition (*p* = 0.046). No difference was found between moderately and severely malnourished patients (*p* = 0.596). Among all three patient groups physical activity was comparable to healthy controls (Supplementary Table 3).

**TABLE 6:** Reported physical activity levels of patients with chronic pancreatitis stratified by nutritional status.

	No malnutrition (*n* = 24)	Moderate malnutrition (*n* = 14)	Severe malnutrition (*n* = 28)	
**Physical activity level**				**†**
Low	7 (29)	1 (7)	5 (18)	
Moderate	10 (42)	3 (21)	8 (29)	
High	7 (29)	10 (71)	15 (54)	

*Differences between groups were tested using Chi-squared test or Fisher’s exact test, respectively.*

*^†^Indicates significant difference between patients without malnutrition and with moderate malnutrition, p < 0.05.*

## Discussion

It is widely accepted that malnutrition is a common complication in CP. However, there is very limited data regarding its causes and consequences.

In this work malnutrition was highly prevalent – affecting almost two-thirds of patients – and in most cases presented in a severe form. Impaired nutritional status resulted from acute episodes of reduced food intake and exacerbations of systemic inflammation. Malnutrition was more commonly seen in patients with initial diagnosis within the last year or higher COPPS. While there was an evident phenotype of severe malnutrition, presentation of moderately malnourished patients was less distinct. Although reduced SMM, even at normal range BMI, presented a characteristic feature of malnutrition, concomitant sarcopenia was rare.

### Risk Factors of Malnutrition

Our results partly contradict what is currently believed to be the relevant determinants of nutritional status in CP. We did not see any association of malnutrition with etiology, continued substance abuse, pain, endocrine or exocrine function. Although, for the most part, contribution of these factors is rather derived from pathophysiological considerations than evidence from clinical trials (22), these findings were unexpected. As almost half of all patients had received their diagnosis within the last year, it is possible that the aforementioned factors increase the risk of malnutrition only with longer disease duration or if treated inadequately. Future trials with a larger cohort of patients with CP are warranted for a definite clarification of an association of these factors with malnutrition.

By contrast, we found a higher prevalence of malnutrition in patients with shorter disease duration or higher COPPS. Weight loss, which was seen in most malnourished patients, is a common symptom leading to hospitalization and will result in subsequent diagnostic work-up (23). This could explain why malnutrition was more common in patients diagnosed within the last year. A higher COPPS has been shown to be associated with an increased risk of readmission and longer hospital stay (15, 24). Because COPPS comprises multiple parameters related to nutritional status and inflammation, our data does not allow to determine whether it is the cause or the effect of malnutrition. However, this association emphasizes the link between malnutrition and adverse clinical outcome and could aid identification of at-risk patients.

Reduced food intake is another factor considered to promote malnutrition in CP. A recent meta-analysis showed that energy intake is not generally lower than in healthy controls (25). In our cohort, malnourished patients more frequently experienced serious acute reduction of food intake. Conversely, higher energy intake, despite significantly lower BMI, was reported for the month preceding hospital attendance as compared to non-malnourished subjects. This finding implies that weight loss in CP rather results from extensive episodically than chronically reduced food consumption. As ratings of acute pain did not differ between groups, impaired dietary intake is likely caused by other factors. We found elevated levels of CRP in severely malnourished patients, which has been linked to reduced food intake in acute disease (26, 27) and could therefore be a relevant mechanism leading to malnutrition in CP.

### Phenotype of Malnutrition

Our results indicate that phenotypic presentation of malnutrition in CP significantly differs depending on its severity. In comparison to non-malnourished patients, subjects with severe malnutrition were characterized by significant weight loss, altered body composition and blood parameters reflecting reduced SMM and elevated inflammation. By contrast, moderate malnutrition was less distinct and primarily indicated by anthropometric and body composition metrics.

Weight loss has repeatedly been observed in patients with CP and related to impaired nutritional status (28–32). While weight loss has commonly been linked to low BMI, a relation to altered body composition in malnourished subjects with CP has only been shown in a single investigation (31). In agreement with our results, this study found lower fat and muscle reserves, indicated by anthropometric measures, in malnourished subjects. Moreover, several studies confirm our findings of a high prevalence of reduced SMM in CP even in the normal to obese BMI range (32–35). While most of these works addressed this condition in the context of sarcopenia, only Verhaegh et al. (32) related their findings to malnutrition. They observed reduced muscle mass in patients with CP compared to healthy controls using both anthropometric and BIA measures and showed that malnutrition screening tools lacked the sensitivity to detect these alterations. Our findings also support that both anthropometric and body composition parameters can detect malnutrition in CP, even at early stage. However, for most parameters there are no established cut-offs and current data are insufficient to derive these values, especially for detection of moderate malnutrition.

Regarding blood parameters indicative of malnutrition, our results point in a similar direction. We found several altered blood markers reflecting reduced SMM and increased systemic inflammation in severe malnutrition.

Systemic inflammation has been linked to impaired nutritional status in CP (36). We found increased levels of CRP in severe malnutrition compared to both non- and moderately malnourished patients, whereas no other inflammatory markers differed. Elevated CRP, in general, is associated with other conditions characterized by muscle wasting such as sarcopenia (37), cachexia (38), and frailty (39). In agreement with our results, a meta-analysis (37) found that levels of CRP but not Interleukin 6 or tumor necrosis factor α were associated with sarcopenia. Although the link between CRP and loss of SMM is mechanistically intriguing, diagnostic value is limited as CRP is generally elevated in patients with CP compared to control subjects (36). Moreover, as an unspecific acute phase reactant with a half-life of less than a day it is unsuitable for detection of malnutrition in a disease of dynamic inflammatory nature (40).

### Muscle Strength and Sarcopenia

A recent meta-analysis (41) found a pooled sarcopenia prevalence of 42% in CP. We detected sarcopenia in only 3% of patients. This markedly lower rate results from the use of different diagnostic criteria. While five out of six studies in this meta-analysis diagnosed sarcopenia solely based on reduced muscle mass, we also assessed muscle strength and physical performance in accordance with current global recommendations on diagnosis (20, 42). We found lowered SMM at a comparable rate (30%) but only a minority of these patients showed concomitant physical impairment. The only other study that included measures of physical performance for diagnosis reported a prevalence of 17% (35), which is more comparable to our findings. Hence, risk of sarcopenia in CP may be substantially overestimated and what is considered sarcopenia in other studies may actually reflect severe malnutrition. Because our analyses are limited by the low prevalence of sarcopenia, it remains subject to further investigation to determine the factors that promote malnutrition and sarcopenia, respectively. However, our findings suggest that there are differences, which could demand for distinct forms of therapy.

### Limitations

There are some limitations to our study. First, we cannot entirely rule out the chance of selection bias. However, regarding underlying etiology and etiologic risk factors our cohort is in agreement with published data. Moreover, retrospective studies have reported comparable findings regarding weight loss and reduced SMM, the predominant criteria leading to malnutrition diagnosis in our study. Second, we measured pancreatic exocrine function by fecal elastase-1 test. Although it is the most widely used test for diagnosis of EPI, it is only reliable in moderate-to-severe EPI (43). Hence, we may have missed early mild forms of exocrine insufficiency. Last, because of its cross-sectional design it is impossible to draw conclusions regarding causal relations. It will be necessary to further investigate the relations we found in a longitudinal design.

## Conclusion

In conclusion, malnutrition is a frequent complication of CP characterized by loss of skeletal muscle mass but rarely concomitant functional impairment. As an impaired nutritional status becomes evident only at an advanced stage, regular testing for altered body composition is recommended. Future research should aim to identify suitable biomarkers of malnutrition in CP and elucidate the link between loss of muscle mass and physical function.

## Data Availability Statement

The datasets presented in this article are not readily available because of ethical and legal considerations. Requests to access the datasets should be directed to the corresponding author.

## Ethics Statement

The studies involving human participants were reviewed and approved by the Local Ethics Committees of University Medicine Greifswald, Rostock University Medical Center, and University of Applied Sciences Neubrandenburg. The patients/participants provided their written informed consent to participate in this study.

## Author Contributions

MW, SG, LV, L-AG, CM, KB, RJ, GL, ML, and AA: conceptualization and methodology. MW: validation, formal analysis, writing – original draft, and visualization. MW, NE, JD, FF, QT, FW, FM, KB, LS, LE, and AS: investigation and data curation. MW, SG, NE, JD, FF, QT, FW, FM, LV, L-AG, CM, KB, LS, LE, RJ, GL, AS, ML, and AA: writing – review and editing. ML and AA: supervision. SG, LV, L-AG, CM, RJ, GL, AS, ML, and AA: funding acquisition. All authors contributed to the work, reviewed and edited the manuscript, and approved the final version of the manuscript.

## Conflict of Interest

The authors declare that the research was conducted in the absence of any commercial or financial relationships that could be construed as a potential conflict of interest.

## Publisher’s Note

All claims expressed in this article are solely those of the authors and do not necessarily represent those of their affiliated organizations, or those of the publisher, the editors and the reviewers. Any product that may be evaluated in this article, or claim that may be made by its manufacturer, is not guaranteed or endorsed by the publisher.

## Abbreviations

BMI: body mass index
COPPS: Chronic Pancreatitis Prognosis Score
CP: chronic pancreatitis
CRP: C-reactive protein
EPI: exocrine pancreatic insufficiency
GLIM: Global Leadership Initiative on Malnutrition
SMM: skeletal muscle mass
SMMI: skeletal muscle mass index.
