# Supplementary material for: Malnutrition Is Highly Prevalent in Patients With Chronic Pancreatitis and Characterized by Loss of Skeletal Muscle Mass but Absence of Impaired Physical Function
Source: Front Nutr. 2022 Jun 1;9:889489. doi: 10.3389/fnut.2022.889489 (PMC9202591; doi:10.3389/fnut.2022.889489)
Supplement: Supplementary file 1 [file Table_1.DOCX]

Supplementary Table 1 Comparison of anthropometric parameters and body composition in patients with chronic pancreatitis stratified by nutritional status and respective healthy controls

|  | No Malnutrition  (n=24) | Control  (n=24) | p-value | Moderate Malnutrition  (n=14) | Control  (n=14) | p-value | Severe Malnutrition  (n=28) | Control  (n=28) | p-value |
| --- | --- | --- | --- | --- | --- | --- | --- | --- | --- |
| Body weight, kg | 92.3 (±11.5) | 77.1 (±12.8) | **< .001** | 77.3 (±9.3) | 82.2 (±12.7) | .259 | 67.8 (±13.7) | 81.6 (±16.1) | **< .001** |
| Waist circumference, cm | 104.9 (±12.7) | 90.1 (±11.8) | **< .001** | 94.5 (±8.0) | 95.2 (±8.7) | .805 | 86.8 (±10.1) | 94.4 (±12.3) | **.015** |
| Hip circumference, cm | 107.8 (±10.8) | 101.8 (±7.9) | **.034** | 97.6 (±4.1) | 102.7 (±5.5) | **.009** | 92.2 (±6.5) | 102.7 (±8.6) | **< .001** |
| Waist-to-Hip ratio | 0.98 (±0.11) | 0.89 (±0.10) | **.003** | 0.97 (±0.06) | 0.93 (±0.07) | .124 | 0.94 (±0.08) | 0.92 (±0.09) | .364 |
| Mid upper arm circumference, cm | 33.1 (±2.7) | 30.5 (±3.1) | **.003** | 28.7 (±2.0) | 31.8 (±3.7) | **.011** | 27.0 (±3.7) | 31.6 (±3.5) | **< .001** |
| Triceps skinfold thickness, mm | 25.2 (±8.4) | 16.9 (±5.4) | **< .001** | 14.5 (±3.5) | 15.9 (±6.8) | .506 | 13.8 (±6.0) | 15.6 (±5.7) | .248 |
| Body mass index, kg/m^2^ | 30.9 (±4.2) | 25.6 (±3.6) | **< .001** | 24.8 (±2.0) | 26.8 (±3.4) | .068 | 22.8 (±3.5) | 26.7 (±4.0) | **< .001** |
| Fat mass index, kg/m^2^ | 11.0 (±4.2) | 7.3 (±2.8) | **< .001** | 6.6 (±1.9) | 7.5 (±1.9) | .187 | 5.7 (±2.3) | 7.8 (±3.1) | **.006** |
| Fat free mass index, kg/m^2^ | 19.9 (±2.0) | 18.3 (±2.1) | **.010** | 18.3 (±1.8) | 19.3 (±2.5) | .218 | 17.1 (±2.8) | 18.9 (±2.4) | **.014** |
| Skeletal muscle mass index, kg/m^2^ | 9.6 (±1.1) | 8.7 (±1.3) | **.013** | 8.5 (±0.7) | 9.2 (±1.5) | .091 | 7.5 (±1.9) | 9.1 (±1.5) | **< .001** |
| Phase angle, ° | 5.3 (±0.7) | 5.3 (±0.6) | .981 | 4.7 (±0.8) | 5.4 (±0.6) | **.017** | 4.4 (±1.0) | 5.4 (±0.7) | **< .001** |
| Total body water, l | 44.4 (±6.3) | 41.0 (±6.8) | .079 | 41.9 (±5.0) | 43.7 (±7.2) | .435 | 37.5 (±8.7) | 42.9 (±7.7) | **.018** |
| Extracellular body water, l | 19.5 (±2.6) | 17.8 (±2.5) | **.021** | 18.6 (±2.8) | 18.8 (±2.8) | .858 | 17.2 (±3.3) | 18.5 (±2.9) | .117 |
| Extracellular to total body water ratio^a^ | 0.43 (0.04) | 0.43 (0.03) | .463 | 0.45 (0.04) | 0.43 (0.02) | .137 | 0.45 (0.05) | 0.43 (0.03) | .**002** |

All data is presented as mean (±SD) unless indicated otherwise

^a^ Data is presented as median (IQR)

Differences between groups were tested using two‑tailed t-test for or Mann-Whitney-U test for normally and non-normally distributed data, respectively
