# Supplementary material for: Malnutrition Is Highly Prevalent in Patients With Chronic Pancreatitis and Characterized by Loss of Skeletal Muscle Mass but Absence of Impaired Physical Function
Source: Front Nutr. 2022 Jun 1;9:889489. doi: 10.3389/fnut.2022.889489 (PMC9202591; doi:10.3389/fnut.2022.889489)
Supplement: Supplementary file 2 [file Table_2.DOCX]

Supplementary Table 2 Comparison of energy and macronutrient intake in patients with chronic pancreatitis stratified by nutritional status and respective healthy controls

|  | No Malnutrition  (n=23)^a^ | Control  (n=23) | p-value | Moderate Malnutrition  (n=14) | Control  (n=14) | p-value | Severe Malnutrition  (n=28) | Control  (n=28) | p-value |
| --- | --- | --- | --- | --- | --- | --- | --- | --- | --- |
| Energy, kcal/d | 1576 (1421) | 1827 (746) | .328 | 2024 (1082) | 1875 (1263) | .511 | 2171 (1002) | 2213 (1114) | .922 |
| Protein, g/d | 59 (43) | 77 (42) | .621 | 83 (36) | 68 (53) | .541 | 78 (34) | 86 (40) | .432 |
| Carbohydrates, g/d | 181 (136) | 182 (80) | .684 | 208 (143) | 165 (137) | .285 | 253 (100) | 228 (182) | .787 |
| Dietary fiber, g/d | 21 (18) | 20 (16) | .660 | 20 (14) | 21 (29) | .571 | 23 (13) | 23 (20) | .306 |
| Fat, g/d | 55 (61) | 66 (44) | .590 | 88 (45) | 66 (40) | .150 | 78 (50) | 74 (40) | .287 |
| Alcohol, g/d | 0 (4) | 9 (11) | **< .001** | 1 (6) | 5 (13) | **.049** | 0 (2) | 7 (13) | **< .001** |

All data is presented as median (IQR)

^a^ One patient did not complete the food frequency questionnaire and was excluded from analysis

Differences between patients with chronic pancreatitis and healthy controls were tested using Mann-Whitney U test
