# Supplementary material for: Malnutrition Is Highly Prevalent in Patients With Chronic Pancreatitis and Characterized by Loss of Skeletal Muscle Mass but Absence of Impaired Physical Function
Source: Front Nutr. 2022 Jun 1;9:889489. doi: 10.3389/fnut.2022.889489 (PMC9202591; doi:10.3389/fnut.2022.889489)
Supplement: Supplementary file 3 [file Table_3.DOCX]

Supplementary Table 3 Reported physical activity levels of patients with chronic pancreatitis stratified by nutritional status and respective healthy controls

|  | No Malnutrition  (n=24) | Control  (n=24) | Moderate Malnutrition  (n=14) | Control  (n=14) | Severe Malnutrition  (n=28) | | Control  (n=28) |  |
| --- | --- | --- | --- | --- | --- | --- | --- | --- |
| **Physical activity level** |  |  |  |  |  | |  |  |
| Low | 7 (29) | 3 (13) | 1 (7) | 3 (21) | 5 (18) | | 7 (25) |  |
| Moderate | 10 (42) | 10 (42) | 3 (21) | 7 (50) | 8 (29) | | 12 (43) |  |
| High | 7 (29) | 11 (46) | 10 (71) | 4 (29) | 15 (54) | | 9 (32) |  |
| **p-value** | .288 | | .103 | | | .268 | | |

Differences between patients with chronic pancreatitis and healthy controls were tested using Chi-squared test or Fisher’s exact test, respectively.
